# Clonal Spread of a Unique Strain of Macrolide-Resistant *Mycoplasma Pneumoniae* Within a Single Family in Italy

**DOI:** 10.1097/MD.0000000000003160

**Published:** 2016-03-18

**Authors:** Maria Chironna, Daniela Loconsole, Anna Lisa De Robertis, Anna Morea, Egidio Scalini, Michele Quarto, Silvio Tafuri, Cinzia Germinario, Mariano Manzionna

**Affiliations:** From the Department of Biomedical Sciences and Human Oncology-Hygiene Section (MC, DL, ALDR, AM, MQ, ST, CG), University of Bari; and Paediatric Unit of the Maternal and Child Health Department of “San Giacomo” Hospital of Monopoli (ES, MM), Bari, Italy.

## Abstract

Macrolide-resistant *Mycoplasma pneumoniae* (MR-MP*)* is an increasing problem worldwide. This study describes the clonal spread of a unique strain of MR-MP within a single family.

On January 23, 2015, nasopharyngeal swabs and sputum samples were collected from the index case (a 9-year-old girl) in southern Italy. The patient had pneumonia and was initially treated with clarithromycin. MR-MP infection was suspected due to prolonged symptoms despite appropriate antibiotic therapy. Two further cases of pneumonia occurred in relatives (a 7-year-old cousin and the 36-year-old mother of the index case); therefore, respiratory samples were also collected from other family members. Sequence analysis identified mutations associated with resistance to macrolides. Both P1 major adhesion protein typing and multiple loci variable-number tandem repeat analysis (MLVA) typing were performed to assess the relatedness of the strains.

The index case, the cousin, the mother, and another 4 family members (twin siblings of the index case, a 3-year-old cousin, and the grandmother) were positive for MR-MP. All strains harbored the mutation A2063G, had the same P1 subtype (1), and were MLVA (7/4/5/7/2) type Z. In addition, the index case's aunt (31 years of age and the probable source of infection) harbored an *M pneumoniae* strain with the same molecular profile; however, this strain was susceptible to macrolides.

This cluster of MR-MP infection/carriage caused by a clonal strain suggests a high transmission rate within this family and highlights the need for increased awareness among clinicians regarding the circulation of MR-MP. Novel strategies for the treatment and prevention of *M pneumoniae* infections are required.

## INTRODUCTION

*Mycoplasma pneumoniae* is a common cause of respiratory tract infections, which can vary from mild upper respiratory tract disease to tracheobronchitis or severe interstitial pneumonia.^1,2^ Furthermore, extrapulmonary complications have been described, which mainly involve the skin, hematologic, cardiovascular, musculoskeletal, and nervous systems.^3,4^ The presence of macrolide-resistant strains and the development of macrolide resistance due to antimicrobial pressure were first reported in Italy during the *M pneumoniae* epidemic of 2010.^5^ During this epidemic the prevalence of macrolide-resistant *M pneumoniae* reached 26%; however, no data regarding the prevalence prior to 2010, nor after the epidemic, have been published. In Europe, the situation varies from country to country, but in general the prevalence of resistant strains is not higher than 10%.^5,6^ The administration of ciprofloxacin as a second line antibiotic in paediatric cases of serious infection with subsequent failure of clarithromycin treatment has proved to be a valid strategy for clinical management.^7^ Multiple locus variable-number tandem repeat analysis (MLVA) is a molecular method based on the sequencing of 5 selected loci; this technique can better discriminate between different strains of *M pneumoniae* than other typing methods and is therefore a useful tool for assessing relationships between cases during an outbreak.^8,9^

Here, MLVA and P1 adhesion protein typing revealed the clonal spread of a unique strain of macrolide-resistant *M pneumoniae* (MR-MP) within a single family in Italy.

## METHODS

### Case Reports

On January 21, 2015, an otherwise healthy 9-year-old girl (index case) with an unremarkable medical history was admitted in the Paediatric Unit of the Maternal and Child Health Department of “San Giacomo” Hospital of Monopoli (Bari, southern Italy) with a diagnosis of pneumonia. In the 11-day period prior to hospitalization, she suffered from a dry cough and fever (39.5°C) accompanied by malaise. She received clarithromycin (prescribed by the family paediatrician) from the second day of symptom onset. She was severely ill upon admission, with a high temperature, dyspnoea, and an increased respiratory rate. Routine blood tests revealed neutrophilia, a high erythrocyte sedimentation rate, and increased C-reactive protein levels. Auscultation revealed diffuse crackles with reduced vesicular sounds on the right lung, and chest radiography showed a right middle lobe infiltrate. Blood, nasopharyngeal swab (NPS), and sputum samples were collected for serological tests and real-time polymerase chain reaction (PCR) analysis of bacterial agents (i.e., those responsible for community-acquired pneumonia) and respiratory viruses. The girl received nebulized albuterol and clarithromycin (15 mg/kg/day, in 2 oral doses). Microbiological tests were negative for respiratory viruses (respiratory syncytial virus, adenovirus, influenza viruses, parainfluenza viruses, human metapneumovirus, human bocavirus, and coronaviruses) and negative for *Streptococcus pneumoniae*, *Haemophilus influenzae*, *Staphylococcus aureus*, *Chlamydia pneumoniae*, *Legionella pneumophila*, *and Moraxella catharralis*. However, PCR identified NPS and sputum samples as positive for *M pneumoniae* DNA. Serological tests were positive for anti-*M pneumoniae* IgM antibodies, but negative for anti-*M pneumoniae* IgG antibodies.

Despite appropriate antibiotic therapy, the girl remained severely ill and MR-MP infection was suspected. On Day 6 after hospitalization, real-time PCR and melting peak analysis identified an MR-MP strain harboring mutations A2063G/A2064G^10^ in NPS and sputum samples taken on the second day of hospitalization; therefore, clarithromycin was replaced by ciprofloxacin (30 mg/kg/day in 2 oral doses). The patient's clinical condition improved rapidly and she was discharged 15 days after admission.

On February 3, 2015, a 7-year-old cousin of the index case, who had a fever (39.0°C) and a cough for 2 weeks, was admitted to the same hospital with a diagnosis of pneumonia. Prior to admission, he had received ceftriaxone for 6 days, followed by clarithromycin. Chest radiography confirmed pneumonia and microbiological analysis of NPS and sputum samples confirmed the presence of *M pneumoniae*. Epidemiological investigation confirmed close contact with the index case.

On February 10, 2015, the 36-year-old mother of the index case was admitted to the same hospital with a diagnosis of pneumonia after the onset of fever and bronchitis at the end of January. *M pneumoniae* was identified in bronchial aspirate samples taken after admission.

Given the epidemiologic link between these cases, active surveillance was set up to identify other cases/carriers of *M pneumoniae* among family members. NPS and blood samples were taken from 7 other family members on February 23, 2015, 5 of whom were positive for *M pneumoniae* (the father of the index case and the father of the 7-year-old cousin tested negative); thus 8 family members in total tested positive for *M pneumoniae*. All subjects provided written informed consent in accordance with Italian and Institutional standards and the principles set down in the Declaration of Helsinki.

DNA was extracted from respiratory samples using a MagnaPure automated extraction system (Roche Diagnostics, Milan, Italy) and pretested in a commercial real-time PCR assay (Arrow Diagnostics, Genoa, Italy) to detect *M pneumoniae*. *M pneumoniae*-positive samples were then tested for macrolide resistance as previously described.^10^ A PCR assay followed by direct amplicon sequencing was developed to detect point mutations in the *M pneumoniae* 23S rRNA gene, which confer resistance to macrolides.^5^ Nested PCR and sequencing were performed for molecular typing of the P1 major adhesion protein; MLVA typing was also performed.^11,12^ *M pneumoniae*-specific IgM and IgG antibodies in serum samples were measured in an enzyme-linked immunosorbent assay.

## RESULTS AND DISCUSSION

The clinical data and the results of serological and molecular tests on samples from the *M pneumoniae*-positive family members (n = 8) are shown in Table 1. No other respiratory tract pathogens were found. Three cases (the grandmother, the second twin sibling, and the aunt of the index case (Table 1)) were asymptomatic at the time of clinical sampling, although the aunt (the mother of the 2 cousins) reported having a cough for several days in December 2014. The aunt was the only family member to harbor an *M pneumoniae* strain susceptible to macrolides, although the molecular profile (P1 subtype and MLVA type) was identical to that of other strains identified in other family members. All subjects, except the grandmother, were positive for anti-*M pneumoniae* IgM antibodies. The A2063G mutation was detected in all patients. Two of the 3 patients with pneumonia were initially treated with clarithromycin, but this was later shifted to ciprofloxacin. The 7-year-old cousin was treated with ceftriaxone, followed by clarithromycin and azithromycin, and recovered completely after 3 weeks.

**TABLE 1:**
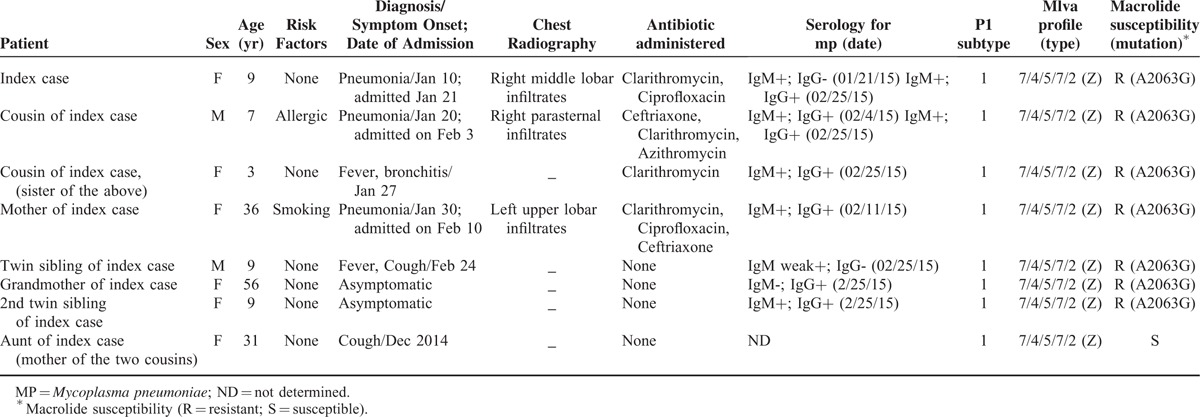
Clinical and Laboratory Findings of Family Members Who Tested Positive for *M Pneumoniae*

Many studies report that MR-MP has spread worldwide since the 2000s. The circulation of MR-MP in Italy was first established in 2010^5,7^ and has since been confirmed in subjects with *M pneumoniae* infection (unpublished data).

MVLA confirmed that a recent outbreak of *M pneumoniae* (macrolide-susceptible strains) in a French primary school was caused by a single clonal strain.^8^ In addition, a familial cluster of atypical pneumonia caused by macrolide-susceptible *M pneumoniae* was reported in South and North Carolina,^13^ and an epidemiological study reported another cluster of MR-MP infections,^14^ although the relatedness of the strains in this cluster was not assessed.

Here, we report a familial cluster of MR-MP infections involving a significant number of subjects. The presence of the same MLVA type and P1 subtype provides evidence of the clonal spread of a unique strain of *M pneumoniae* among several members of the same family. Based on these findings, we can hypothesize that the index case transmitted the MR-MP infection to her cousin (the 7-year-old male), mother, and twin siblings. The 7-year-old cousin probably transmitted the infection to his 3-year-old sister, who subsequently developed bronchitis. However, it is difficult to explain the presence of a macrolide-susceptible strain of *M pneumoniae* in samples from the index case's aunt, despite the finding that the strain had the same MLVA type and P1 subtype as those of strains identified in other family members. We speculate that the aunt was infected or colonized by a macrolide-susceptible *M pneumoniae* strain in December 2014, and that she transmitted this strain to her niece (the index case); the strain then developed resistance to macrolides following treatment with clarithromycin. The index case then transmitted the infection to her 7-year-old cousin. All other cases were infected subsequent to this. Carriage may be supposed in the case of the grandmother, since she was asymptomatic.

In humans, the acquisition of a macrolide-resistant mutation may occur during the course of infection;^5,15–17^ also, macrolide-resistant mutants originate from wild-type *M pneumoniae.*^18^ In addition, *M pneumoniae* can be present at the nasopharyngeal level for many months;^16,19^ this may be why the aunt of the index case was still positive at the time of sampling. Taken together, the findings reported in these previous studies may help to explain the cluster of infection/carriage within a single family.

Two of the household members who were positive for MR-MP had no respiratory symptoms and never developed disease. This, to the best of our knowledge, is novel since carriage of *M pneumoniae* DNA has been reported only in subjects infected by strains who are susceptible to macrolides;^8^ to date, there are no reports describing carriage of macrolide-resistant strains.

In conclusion, this study reports the spread of a unique clonal MR-MP strain within a single family in Italy. The presence of macrolide-resistant strains in the pharyngeal tracts of asymptomatic family members highlights the potential for such subjects to spread macrolide-resistant strains in the community. These findings underline the need for routine identification of MR-MP and increased awareness among clinicians regarding such occurrences. Finally, increasing resistance to macrolides means that novel strategies for treating and preventing *M pneumoniae* infections should be considered.

## References

[1] Waites KB Talkington DF *Mycoplasma pneumoniae* and its role as a human pathogen. *Clin Microbiol Rev* 2004; 17:697–728. 15489344 10.1128/CMR.17.4.697-728.2004 PMC523564

[2] Principi N Esposito S Macrolide-resistant *Mycoplasma pneumoniae*: its role in respiratory infection. *J Antimicrob Chemother* 2013; 68:506–511. 23169891 10.1093/jac/dks457

[3] Atkinson TP Waites KB *Mycoplasma pneumoniae* infections in childhood. *Pediatr Infect Dis J* 2014; 33:92–94. 24346598 10.1097/INF.0000000000000171

[4] Narita M Pathogenesis of extrapulmonary manifestations of *Mycoplasma pneumoniae* infection with special reference to pneumonia. *J Infect Chemother* 2010; 16:162–169. 20186455 10.1007/s10156-010-0044-x

[5] Chironna M Sallustio A Esposito S Emergence of macrolide-resistant strains during an outbreak of *Mycoplasma pneumoniae* infections in children. *J Antimicrob Chemother* 2011; 66:734–737. 21393214 10.1093/jac/dkr003

[6] Peuchant O Ménard A Renaudin H Increased macrolide resistance of *Mycoplasma pneumoniae* in France directly detected in clinical specimens by real-time PCR and melting curve analysis. *J Antimicrob Chemother* 2009; 64:52–58. 19429926 10.1093/jac/dkp160

[7] Cardinale F Chironna M Dumke R Macrolide-resistant *Mycoplasma pneumoniae* in paediatric pneumonia. *Eur Respir J* 2011; 37:1522–1524. 21632830 10.1183/09031936.00172510

[8] Pereyre S Renaudin H Charron A Clonal spread of *Mycoplasma pneumoniae* in primary school, Bordeaux, France. *Emerg Infect Dis* 2012; 18:343–345. 22305177 10.3201/eid1802.111379 PMC3310468

[9] Chalker VJ Pereyre S Dumke R International *Mycoplasma pneumoniae* typing study: interpretation of *M pneumoniae* multilocus variable-number tandem-repeat analysis. *New Microbes New Infect* 2015; 7:37–40. 26236493 10.1016/j.nmni.2015.05.005 PMC4501435

[10] Dumke R von Baum H Lück PC Occurrence of macrolide-resistant *Mycoplasma pneumoniae* strains in Germany. *Clin Microbiol Infect* 2010; 16:613–616. 19765022 10.1111/j.1469-0691.2009.02968.x

[11] Dumke R Lück PC Noppen C Culture-independent molecular subtyping of *Mycoplasma pneumoniae* in clinical samples. *J Clin Microbiol* 2006; 44:2567–2570. 16825381 10.1128/JCM.00495-06 PMC1489489

[12] Dumke R Jacobs E Culture-independent multi-locus variable-number tandem-repeat analysis (MLVA) of *Mycoplasma pneumoniae*. *J Microbiol Methods* 2011; 86:393–396. 21704086 10.1016/j.mimet.2011.06.008

[13] Rhea SK Cox SW Moore ZS Centers for Disease Control and Prevention (CDC). Notes from the field: atypical pneumonia in three members of an extended family—South Carolina and North Carolina, July–August 2013. *MMWR Morb Mortal Wkly Rep* 2014; 63:734–735. 25144546 PMC5779437

[14] Tsai V Pritzker BB Diaz MH Cluster of macrolide-resistant *Mycoplasma pneumoniae* infections in Illinois in 2012. *J Clin Microbiol* 2013; 51:3889–3892. 23966493 10.1128/JCM.01613-13 PMC3889792

[15] Itagaki T Suzuki Y Seto J Two cases of macrolide resistance in *Mycoplasma pneumoniae* acquired during the treatment period. *J Antimicrob Chemother* 2013; 68:724–725. 23152478 10.1093/jac/dks454

[16] Nilsson AC Jensen JS Björkman P Development of macrolide resistance in *Mycoplasma pneumoniae*-infected Swedish patients treated with macrolides. *Scand J Infect Dis* 2014; 46:315–319. 24447251 10.3109/00365548.2013.866268

[17] Averbuch D Hidalgo-Grass C Moses AE Macrolide resistance in *Mycoplasma pneumoniae*, Israel, 2010. *Emerg Infect Dis* 2011; 17:1079–1082. 21749775 10.3201/eid1706.101558 PMC3358208

[18] Dumke R Stolz S Jacobs E Molecular characterization of macrolide resistance of a *Mycoplasma pneumoniae* strain that developed during therapy of a patient with pneumonia. *Int J Infect Dis* 2014; 29:197–199. 25449256 10.1016/j.ijid.2014.07.014

[19] Meyer Sauteur PM van Rossum AM Vink C *Mycoplasma pneumoniae* in children: carriage, pathogenesis, and antibiotic resistance. *Curr Opin Infect Dis* 2014; 27:220–227. 24751894 10.1097/QCO.0000000000000063

